# The Practical Problem With Carbapenem Testing and Reporting Accurate Bacterial Susceptibilities

**DOI:** 10.3389/fphar.2022.841896

**Published:** 2022-04-25

**Authors:** Mark Redell, Glenn S. Tillotson

**Affiliations:** ^1^ Melinta Therapeutics, Morristown, NJ, United States; ^2^ GST Micro LLC, North Virginia, VA, United States

**Keywords:** breakpoints, susceptibility, antimicrobial susceptibility test, carbapenem-resistant Enterobacterales, clinical outcomes

## Abstract

**Background:** Antibiotic resistance is an evolving issue which requires constant review. Susceptibility breakpoints are revised in line with new microbiological and pharmacological data. Susceptibility breakpoints for carbapenems and Enterobacterales were revised in response to the rise in resistance and the potential for standard doses of carbapenems to provide the necessary antibiotic exposure and to accurately identify rates of carbapenem resistance.

**Objectives:** This review sought to identify real-world implications associated with lack of testing and reporting current carbapenem breakpoints and potential barriers that may impede implementation of these strategies.

**Methods:** A literature review was conducted using PubMed and Google Scholar electronic databases.

**Results:** The failure to adopt revised breakpoints incurs negative clinical outcomes and carries increased cost implications. However, there were several impediments highlighted which are barriers for laboratories to implement breakpoint updates.

**Conclusion:** Possible practical steps to implement revised breakpoints which apply to carbapenems and Enterobacterales are proposed. The challenge for laboratories is to be aware and implement these changes to provide accurate and relevant susceptibility results for clinicians.

## Introduction

Both the susceptibility interpretation and the antibiotic dose relative to the pathogen’s minimum inhibitory concentration (MIC) help predict clinical and microbiological outcomes, although patient-specific characteristics such as body weight and renal and hepatic function are incorporated into the final dosage regimen decision. Appropriate drug selection and administered dosage are defined both in the U.S. Food and Drug Administration (FDA) label, specific to approved disease state(s), and guidance from standards development organizations (SDOs) such as the FDA, Clinical Laboratory Standards Institute (CLSI), and European Committee on Antimicrobial Susceptibility Testing (EUCAST) are critical in establishing and re-evaluating microbiological breakpoints.

While susceptibility breakpoints are blunt and fixed instruments in assessing chances for positive clinical outcomes, drug exposures create another under-appreciated dimension. For most common antibiotics used in critically ill patients, a clear therapeutic range has been established, and for these agents, routine TDM in such patients appears valuable. Unfortunately, TDM is performed by few centers and has not widely impacted clinical practice but holds future promise (Abdul-Aziz et al., 2020). Drug exposures will vary between patients even when the same dose regimen is administered. These pathogen-specific (i.e., MIC value and breakpoint) and patient-specific (i.e., drug exposure at site of infection) characteristics co-exist to provide clinicians the best means for applying the principles of precision medicine (Bader et al., 2018).

Besides assignment of susceptibility interpretations and appropriate dosages, introduction of a novel antibiotic to the marketplace faces another difficult set of challenges. Surveillance studies which compare a new investigational agent against marketed comparators may only extend the comparisons to *in vitro* potency based upon MICs. A more immediate challenge for a new drug approved for clinical use is the delay in approved susceptibility testing devices from manufacturers. The FDA sets specific susceptibility breakpoints for new drugs and device manufacturers finalize testing platforms based on these MICs which creates this delay. “New drugs without tests” (Humphries and Hindler, 2016) continues to reverberate throughout laboratories. The lag in time can be appreciable yet FDA Guidance has attempted to facilitate use of an improved model focusing on sponsor-manufacturer pre-approval planning (US FDA, 2019). A new guidance from the FDA facilitates the 510k process and suggests sponsors of a new agent coordinate with antimicrobial susceptibility testing (AST) device development and clearance according to an algorithm and which can be initiated prior to FDA review of the new drug. Recently the FDA has cleared three AST devices within 33–41 days after a 510k submission and 44 days after drug approval (US FDA, 2019). Finally, adoption of a new antibiotic into clinical practice requires the laboratory to perform verification studies on AST devices prior to the testing and reporting of AST results for the new antibiotic. In addition, laboratories participate in proficiency testing programs to ensure the accuracy and precision of their laboratory performance.

## Why Revise Breakpoints?

Over time, the evolution and distribution of microbial resistance and resistance determinants necessitates re-assessment and revision of breakpoints for several antibiotic classes. Recent changes in breakpoints attempt to improve the scientific process of linking susceptibility, resistance (including species-specific shifts in wild-type distribution of MICs), antibiotic dosing, and clinical outcomes. With these complex interacting pieces, pharmacokinetic/pharmacodynamic analyses test whether FDA-approved dosage regimens provide target levels of drug exposures that are associated with bacterial killing *in vivo* for organisms with MICs at and below the selected susceptibility breakpoint (Dudley, 2012). An excellent summary of CLSI breakpoint revisions since 2010 for aerobic bacteria has been provided (Humphries et al., 2019). New mathematical approaches have fostered an industry of experts and SDO which apply pharmacodynamic principles to justify or revise antibiotic breakpoints (Satlin et al., 2020a; Satlin et al., 2020b).

Breakpoint revisions add to the complexity of verification studies as laboratories can be over-whelmed with incorporation of new breakpoints into their daily testing routines. The incorporation of revised breakpoints into laboratory practice remains an important issue only partly solved by the 21st Century Cures Act and reflects more on regulatory processes and the role of AST device manufacturers (Humphries et al., 2018a). This commentary sought to identify the impact associated with a lack of AST testing and reporting of current carbapenem breakpoints as well as identify potential barriers that may impede laboratory implementation of AST testing and reporting.

## Methods

A literature search was conducted using the PubMed and Google Scholar electronic database to identify established breakpoints, institutional guidelines as well as investigate clinical outcomes associated with non-reporting of current breakpoints. The following search terms were used: “antibiotic breakpoints,” “antibiotic susceptibility testing,” “CARES Act,” “carbapenem breakpoints,” “antibiotic manufacturers,” “breakpoint revisions,” “CLSI breakpoints,” “EUCAST breakpoints,” “microbiology laboratories,” “carbapenem MIC,” and “breakpoint revisions”. Additionally, FDA, CLSI and EUCAST websites and databases were utilized in a secondary search to determine current regulatory requirements and clinical microbiology laboratory protocols and practices.

## Results

### Extent of Using Historic Carbapenem Breakpoints for Enterobacterales in Clinical Laboratories

The Centers for Disease Control and Prevention (CDC) analyzed clinical microbiology laboratory practices for detection of multi-drug-resistant (MDR)-Enterobacterales in almost 5,000 acute care hospitals (2015 and 2016 calendar years) participating in the National Healthcare Safety Network (NHSN) Patient Safety Component Annual Hospital Survey. The survey found that in 2016, 1,063 hospitals (23%) used CLSI pre-2010 MIC interpretive criteria for detecting CRE (Shugart et al., 2018) and of these participants, 464 hospitals (44%) reported not testing for carbapenemases. During this same period, California hospitals were surveyed (fall 2015 to spring 2016) regarding use of current CLSI carbapenem breakpoints for Enterobacterales (Humphries et al., 2018b). The authors found 72% (92/128) of responding laboratories had incorporated the revised CLSI breakpoints into their practices, however time to implementation was up to 68 months (mean, 41 months; median, 55 months). Similar to the CDC findings, 40% of laboratories using historical CLSI breakpoints did not perform carbapenemase testing on site. Recently results from the 2019 College of American Pathologists, an accrediting organization for laboratories based in the U.S., of 982 respondents, only 62.1% incorporated FDA or CLSI breakpoints or disk diffusion zones into their susceptibility test devices (Simner et al., 2022).

EUCAST has tracked implementation of breakpoints for several years throughout Europe. A report follows the progress to April 2019 (EUCAST, 2019) on adoption of EUCAST breakpoints and guidelines, with most countries incorporating National AST Committees. An earlier survey by Brown et al. (2015) shows progress up to 2013.

An online survey conducted in August 2019 (Data on file Melinta Therapeutics, 2019) assessing revised carbapenem breakpoint implementation was sent through the American College of Clinical Pharmacy (ACCP) Infectious Diseases Research Network. Responses were obtained from 20 U.S. institutions from 8 of 10 CDC regions and primarily represented general care hospitals of >200 beds. Results showed implementation of revised carbapenem breakpoint occurred in 13 of 20 (65%) separate microbiology laboratories. While the number of respondents was small, these results are similar to findings by Humphries et al. which reported 72% (92/128) of laboratories in California had updated breakpoints as of July 2017. Despite all 20 sites claiming to have adopted at least 5 of 7 CDC/TJC mandated stewardship Core Elements (CDCP, 2014), the above findings may be evidence that breakpoint changes are not communicated to members of the ASP by microbiology lab personnel.

Most recently, a proficiency testing survey conducted by the College of American Pathologists in June 2019 and replies from 982 US clinical laboratories demonstrated that only 62.1% implemented current breakpoints for meropenem and Enterobacterales (Simner et al., 2022). Compared to 187 ex-US laboratories, 79.7% incorporated current breakpoints for meropenem (*p* < 0.001). Despite use of obsolete breakpoints overall, 13% were unaware of breakpoint changes or the need to update these and 55.9% had no plans to update to current standards. Laboratories continue to struggle with incorporating revised breakpoints into daily clinical practice. While most antibiotic breakpoints are identical between the FDA and CLSI, confusion will exist when breakpoints are not synchronized. Humphries et al. provide none examples of recent CLSI breakpoints not recognized by the FDA (Humphries et al., 2019). The FDA STIC website provides a quick access resource for laboratories and synchronized breakpoints are noted by a comment “FDA recognizes [CLSI] M100 breakpoints” (FDA STIC, FDA Antibacterial Susceptibility Test Interpretive Criteria, 2020). However, there is no practical guidance for selecting one set of breakpoints over the other. The urgency of converting to the new revised breakpoints has varied in the past but efforts are underway to resolve some of these discrepancies. Finally, AST devices are cleared by the FDA thus manufacturers are obliged to follow breakpoints from this body.

### Quantification of Isolates With MICs Between Historic and Current Breakpoints

Investigators tested over 10,000 isolates of Enterobacterales and compared susceptibilities between historic (CLSI M100-S19) and more recent (CLSI M100-S29) breakpoints (Yarbrough et al., 2020). For meropenem using current breakpoints as the reference standard, the very major error (VME) rates for all Enterobacterales isolates, blaKPC carbapenemase-producing CRE, and blaKPC isolates, were 45, 30, and 28%, respectively. Other investigators demonstrated that 22.9% (25/109) of carbapenemase-producing CRE had meropenem MICs ≤4 mg/L and 18.3% (20/109) showed MICs of 2 and 4 mg/L (Tamma et al., 2016). These results are very similar to those observed at the University of California, Los Angeles (Humphries et al., 2018b) where about 20% of carbapenemase producers would be interpreted as susceptible using historical breakpoints compared to only 1–3% using revised breakpoints.

### Implications of Not Using Revised Breakpoints

Failure to adopt revised breakpoints may incur several negative quality and clinical outcomes. These have been addressed recently (Redell and Tillotson, 2019). The failure to harmonize breakpoints between laboratories has led to inconsistent and inaccurate clinical reporting of antimicrobial susceptibility and compromises the quality of data emanating from the laboratory. Most importantly this results in the inability to track pathogen resistance patterns across geographic regions.

#### Increased Errors in Reporting

Erroneously calling a non-susceptible bacterial isolate “susceptible” is referred to as a VME. This is the most undesirable reporting error for clinical microbiology laboratories and reflects lack of concordance with quality guidelines. Failure to update breakpoints in the laboratory neglects the importance and processes of validation despite its burden (Humphries and Simner, 2020; Wojewoda et al., 2020; Kirby et al., 2019). Since calculation of VME integrates the resistant subpopulation and not the total number of isolates as the denominator, percent VME rates can be appreciable (CLSI, 2015). Castanheira et al. (2017) examined the MIC distribution of meropenem in 265 isolates of Enterobacterales identified as CRE. The number of isolates with meropenem MICs ≤1 mg/L and ≤4 mg/L were 5 (1.9%) and 69 (26.0%), respectively, demonstrating the effective exclusion of isolates using the current breakpoints. In addition, of 135 isolates of Enterobacterales identified as KPC-producing and using the above MIC cut-offs, only 1 (0.7%) isolate versus 18 (13.3%) isolates would be reported as CRE. Therefore, laboratories that use revised carbapenem breakpoints detect significantly more carbapenem- and cephalosporin-resistant Enterobacterales compared to laboratories that use historical breakpoints. Clinical use of a carbapenem to treat an infection due to carbapenemase-producing Enterobacterales, despite a phenotype of ‘susceptible’, highlights a potential *in vitro*—*in vivo* discordance which could adversely affect outcomes. In such circumstances of discordance, laboratories should repeat susceptibility testing.

Bacterial isolates whose true MIC value is near the breakpoint may contribute to VMEs. The CLSI M52 recommends a rate of VME of ≤1.5% based upon the resistant population. In a collection of isolates in which a substantial number test at MICs that lie at the ends of and in between historic and revised MICs the rate of VMEs is expected to increase as a function of breakpoints “shifting to the left” (CLSI, 2015). This has been shown to be the case for carbapenems and cephalosporins – reliance on older breakpoints for Enterobacterales has led to discrepant and erroneous susceptibility interpretation (Heil and Johnson, 2016; Yarbrough et al., 2020).

#### Clinical Outcomes and Resources Suffer

There have been no formal analyses or estimates of the economic burden comparing infections due to Enterobacterales with MICs ≤1 mg/L (current) versus MICs >1 mg/L (historic). Costs associated with use of outdated breakpoints for identifying CRE largely comes from the deleterious effects of delaying appropriate therapy. Inappropriate antibiotic therapy increases the following outcomes: morbidity and mortality, hospital resource utilization, patient length of stay, days of antibiotic therapy, discharge to a long-term care facility, and competition for use of ancillary equipment (i.e., ventilators and infusion pumps). Prolonged therapy with inappropriate antimicrobials can result in increased hospital-associated infections with *Clostridioides difficile*, vancomycin-resistant *Enterococcus faecium*, and *Candida* spp. from prolonged selective pressure (Kang et al., 2005; Lodise et al., 2019; Redell and Tillotson 2019). Competition for resources is intensified which may already be negatively impacted by the COVID-19 pandemic. Furthermore, inappropriate antibiotic usage leads to the necessity for additional therapeutic agents to be added to a patient’s regimen, prolongs the number of days of therapy, and unnecessarily increases the exposure to nephrotoxic agents (Nicasio et al., 2010). In a recent study, only 45% of patients with CRE infections received a microbiologically active antibiotic within 3 days of index culture and those who failed to receive appropriate therapy had a 2-fold increase in in-hospital cost (Lodise et al., 2019).

Misclassification of CRE as carbapenem-susceptible ensures spread of CRE by 3–5% annually (Bartsch et al., 2016; McKinnell et al., 2019). An invisible source of additional CRE infection is represented by CRE carriers as these microbes are transmitted horizontally to induce a state of colonization. Active surveillance, such as screening for colonization, can be limited by resources and costs but in a targeted epidemiological investigation can uncover the source(s) for MDROs. The results could be to further limit transmission throughout hospital wards and to other facilities where patients are transferred. Active surveillance programs are facilitated by increasing use of molecular assays to quickly identify carbapenemase-producing organisms (Simner et al., 2016). Ultimately, colonization with CRE could manifest as outbreaks in compromised patients (McKinnell et al., 2019). Using a simulation model, Bartsch and others found that a 32-month delay in changing carbapenem breakpoints resulted in 1,821 additional CRE carriers—an outcome that could have been avoided by identifying CRE and initiating the appropriate isolation or contact precautions. The authors suggest that a policy aimed at minimizing delay in the adoption of new breakpoints for antimicrobials against emerging pathogens should be implemented when the containment of spread is paramount (Bartsch et al., 2016).

MICs can be linked to human clinical outcomes. To identify a carbapenem clinical breakpoint predicting mortality from Enterobacterales infections, one group found meropenem or imipenem MICs of 2–8 mg/L were significantly associated with an increased 30-day mortality (38.9 vs. 5.6%, *p* = 0.04) and longer lengths of stay in the intensive care unit (ICU) (56.5 versus 21.7 days, *p* < 0.01) compared to patients with susceptible MICs using the new breakpoints (Patel and Nagel, 2015; O’Donnell et al., 2016). A study by Biehle et al. (2015) of 107 patients treated for bloodstream infections due to *Klebsiella pneumoniae* found that mortality was significantly associated with imipenem or meropenem MICs >1 mg/L (OR 9.08; 95% CI 1.17–70.51) after controlling for a variety of factors. As a result of the interaction between mortality and elevated carbapenem MICs, use of revised carbapenem breakpoints enables more accurate estimates of patient survival risk and can be incorporated into risk-adjusted models.

### Barriers to Implementation of Revised Breakpoints

We recognize that implementing revised breakpoints may be challenging as it impacts laboratory workflow and management of technical staff needed to perform analytical validation studies. Enforcement of CAP-accredited laboratories to either adopt a methodology that is up to date or validate their current systems for updated breakpoints (both MIC and disk diffusion test results) using FDA, CLSI or EUCAST criteria must be accomplished by 1 January 2024 (Simner et al., 2022). An institution’s review of breakpoints from three SDOs will encounter further complications with implementation of revised breakpoints and should be discussed with the antibiotic stewardship team. An example is applying EUCAST’s susceptible breakpoint of meropenem for Enterobacterales of ≤0.001 mg/L or disk zone ≥50 mm (Meylan and Benoit, 2020). Differences in susceptible breakpoints between SDOs extend to many other antibiotics, such as polymyxins and cefiderocol.

The CDC provides several bacterial challenge sets for purposes of verification which can be used by laboratories to calculate categorical agreement (CA) and essential agreement (EA) (FDA/CDC AR Isolate Bank). Unfortunately, many challenge sets consist of bacterial isolates with MICs at the extremes of susceptible and resistant ranges which may predictably lead to nearly 100 percent CA and EA. When breakpoints are revised and differ from historic ones by one to two dilutions, combined with many clinical isolates with MICs within this window, the laboratory’s ability to differentiate susceptible, intermediate, and resistant becomes prone to major errors. Unfortunately, most carbapenems for which revised breakpoints were issued several years ago may not be included in automated susceptibility testing panels and laboratories must rely on AST manufacturers to develop and commercialize panels incorporating new breakpoints rather than undergoing verification studies with existing instruments.

### What Facilitates Needed Changes in Implementing Revised Breakpoints?

There is less interest amongst laboratories in revision of carbapenem breakpoints in the absence of a state, federal, or regulatory body mandate for reporting carbapenem-resistant Enterobacterales*.* A significant minority (23%) (Shugart et al., 2018) of US-based laboratories, as of 2016, had not implemented revised carbapenem breakpoints. This has delayed implementation of readily available tools which can guide laboratories through the steps of validating revised breakpoints (Patel et al., 2013). Verification rules established by the College of American Pathologists and Clinical Laboratory Improvement Amendments (CLIA) are not concise nor clear. Currently, CLSI’s M52 serves as the standard of practice for validation processes while CLIA provides the regulatory compliance piece (CMMS, 2017). It is important to recognize that verification studies are beyond quality control testing (Kirby et al., 2019; Humphries and Simner, 2020) and the two are not synonymous.

ASPs emphasize the selection of the right drug, at the right dose, and at the right time. ASP teams must work more closely with their microbiology colleagues to avoid situations where false susceptibility might be reported. Best practice models should be established to advise clinical microbiologists and provide solutions which can assist in circumventing inevitable roadblocks. In the end, physicians who act on susceptibility reports should know whether updated breakpoints have been incorporated into the laboratory.

## Discussion

In 2019 an expert panel was convened during a biannual CLSI meeting (Melinta, 2019) which listed five challenges or barriers to the revision of carbapenem (or any antibiotic) breakpoints:

1. Lack of awareness of breakpoint changes. Increased awareness of key resources can provide valuable education and updates to microbiologists and affiliated healthcare professionals. These include updates in the annual CLSI M100 manual and the CDC Antibiotic Resistance Network (FDA/CDC AR Isolate Bank). Accessing the Antibiotic Resistance Isolate Bank and participation in CLSI national webinars (supported by CLSI and Association of Public Health Laboratories) on changes to breakpoints and susceptibility testing methods (as published in the M100 manual) serve as valuable resources.
2. Staffing and financial resources in the laboratory. Validation procedures can be labor-intensive and economically untenable. An AST’s performance can be affected by many factors. However, validation of newly implemented breakpoints can serve as an effective quality assurance program and a check on technical competency (Humphries and Simner, 2020). More importantly many laboratorians do not have guidance as to first steps in assessing workload requirements; both under-estimation and over-estimation assuredly complicate timely delivery of new breakpoint implementation. The most common reason cited by laboratory respondents in the 2019 CAP proficiency testing survey for continued use of obsolete breakpoints was manufacturer-related issues, such as assuming that use of an FDA-cleared AST system is both necessary and sufficient to assure quality results (51.3%), followed by lack of internal resources to perform analytical validation studies (Simner et al., 2022).
3. Prevalence of CRE and formulary positioning of newer antibiotics. Some laboratories and ASPs may perceive that validation and scheduled quality control testing is not worthwhile if there are few CRE isolates generated in the institution’s annual cumulative antibiogram even when applying historic breakpoints. Despite this perception, CRE have been reported in all 50 US states and European countries. In 2014, Puerto Rico reported a 32.9% CRE rate amongst tested Enterobacterales collected from central line-associated bloodstream infections, catheter-associated urinary tract infections and surgical site infections (Centers for Disease Control and Prevention, 2022). By 2019, this rate had fallen to 17.9%.
4. State public health laboratories need to assume a leadership role. Every state health department differs on how it chooses to report CRE and how it educates hospital and reference labs within its regions. Without mandates to submit suspected CRE isolates to state public health or regional CDC laboratories the detection of CRE will be under-estimated. Implementation of revised breakpoints would produce a more accurate statewide assessment of CRE and can better identify resources to limit horizontal spread. A recent example of how a county public health service collaborated with local hospital laboratories has been discussed (McKinnell et al., 2019).
5. External resources are lacking. In general, laboratory directors are not aware of available regional experts. State and public health laboratories can provide a list of resources for microbiologists to use. Some are beginning to be developed and circulated, such as efforts by California’s Antimicrobial Resistance Lab-Epi Alliance to include instructional slides, tools and worksheets (California Department of Public Health, 2022).

Clearly, resources in the form of toolkits which address a validation plan, spreadsheets for recording validation results, educational webinars and newsletters, checklists, and real-world examples are needed. Opportunities identified by experts during this panel discussion included the following:

1. Create momentum and encouragement. States should mandate CRE reporting with clear rules on sending isolates to the State Laboratory. Many labs do not know they have CRE and lack awareness of this public health threat. An innovation at the Los Angeles County Public Health Laboratory has a public health epidemiologist plus an infectious diseases physician who educate on CRE containment, breakpoints, rapid diagnostic testing, reporting MIC distributions, and troubleshooting (McKinnell et al., 2019). One strategy identified by 27.4% of US clinical laboratories, as an alternative to updating breakpoints for institutional use, has been to send specific isolates to reference labs for testing (Simner et al., 2022). However, this results in significant delays of critical information.
2. Centers of Excellence (CoEs) need to be identified. State public health laboratories and academic centers should serve as CoEs in their states, especially when mandating reporting, or at least serve as a resource for troubleshooting. There are several examples of published tools available to microbiologists for developing a plan for validation of revised breakpoints (Van et al., 2019).
3. Increase educational engagement with the Susceptibility Test Manufacturer’s Association (STMA). Device manufacturers use technical representatives (field-based experts) who could serve as educators for their instruments, both hardware and software. These opportunities can occur during installation of updated software into automated susceptibility devices. Technical representatives from these companies can improve educational outreach and provision of additional tools which discuss changes in breakpoints for a variety of antibiotics and pathogens.
4. Improve link with electronic health records and computer interfaces. Vendors or onsite technical representatives for hospital-based IT and LIMS software systems could be approached to co-develop solutions for accurate transfer of microbiology susceptibility data from the lab to the EHR and prescriber.

## Conclusion

Use of appropriate breakpoints is essential to accurate reporting of susceptibilities. From an antibiotic stewardship perspective, the institution of appropriate therapy is compromised when an antibiotic is not expected to behave as predicted because the pathogen is actually resistant. The list of devastating consequences from reporting false susceptibilities include increased morbidity and mortality, loss of infection prevention and control measures, and increased resource utilization. While some institutions may find Enterobacterales isolates which have very low MICs or very high MICs, well-spaced from the MIC range which is impacted the most (e.g., meropenem MIC range of 1 mg/L to 4 mg/L), several studies have shown that potential for reporting false susceptibilities can still be high and unacceptable. We argue that even a VME rate of 1.5% can have significant clinical and public health consequences. ASPs can take a lead role in educating microbiologists and incorporate breakpoint revisions in their agendas.

A graphical process for revised breakpoint implementation on commercial antimicrobial susceptibility test devices and the roles of CLSI, FDA, and manufacturers has been provided (Humphries et al., 2019). Fortunately, toolkits and educational resources are available from a wide array of experts and societies, such as American Society for Microbiology (ASM, 2016) and Infectious Diseases Society of America (IDSA) (Infectious Diseases Society of America, 2022). An instructive video describes implementation of revised carbapenem breakpoints (California Department of Public Health, 2018). We believe that there are many other resources unknown to us that are currently being used to educate microbiologists on the need to proceed with validation of revised carbapenem breakpoints and to inform clinicians that working with their laboratories is essential.

## Abbreviations

AST, antimicrobial susceptibility test; ASP, antimicrobial stewardship program; CA, categorical agreement; CDC, Centers for Disease Control and Prevention; CLIA, Clinical Laboratory Improvement Amendments; CLSI, Clinical Laboratory Standards Institute; CoE, center of excellence; CRE, carbapenem-resistant Enterobacterales; EA, essential agreement; EHR, electronic health record; EUCAST, European Committee on Antimicrobial Susceptibility Testing; FDA, U.S. Food and Drug Administration; IT, information technology; KPC, Klebsiella pneumoniae carbapenemase; LIMS, laboratory information management system; MIC, minimum inhibitory concentration; MDRO, multiple-drug resistant organisms; SDO, standards development organization; TDM, therapeutic drug monitoring; VME, very major error (% VME is defined according to the number of false-susceptible isolates divided by the total number of resistant isolates identified by the reference method).
